# Preeclampsia and severe acute respiratory syndrome coronavirus 2 infection: a systematic review

**DOI:** 10.1097/HJH.0000000000003213

**Published:** 2022-07-22

**Authors:** Giovanni Tossetta, Sonia Fantone, Nicola delli Muti, Giancarlo Balercia, Andrea Ciavattini, Stefano Raffaele Giannubilo, Daniela Marzioni

**Affiliations:** a Department of Experimental and Clinical Medicine, Università Politecnica delle Marche, Umberto I Hospital; b Clinic of Obstetrics and Gynaecology, Department of Clinical Sciences, Università Politecnica delle Marche, Salesi Hospital, Azienda Ospedaliero Universitaria; c Division of Endocrinology, Department of Clinical and Molecular Sciences, Università Politecnica delle Marche, Umberto I Hospital, Ancona, Italy

**Keywords:** angiotensin-converting enzyme-2, coronavirus disease 2019, infection, preeclampsia, pregnancy, severe acute respiratory syndrome coronavirus 2, virus

## Abstract

**Objective:**

Severe acute respiratory syndrome coronavirus 2 (SARS-CoV-2) is the cause of the coronavirus disease 2019 (COVID-19) disease that has rapidly spread worldwide, causing hundreds of thousand deaths. Normal placentation is characterized by many processes strictly regulated during pregnancy. If placentation is impaired, it can lead to gestational disorders, such as preeclampsia that is a multisystem disorder that occurs in 2–8% of pregnancies worldwide.

**Methods:**

We performed a systematic search to understand the potential involvement of SARS-CoV-2 in preeclampsia onset using the databases, PubMed and Web of Science until 31 January 2022.

**Results:**

SARS-CoV-2 infection not only causes damage to the respiratory system but also can infect human placenta cells impairing pivotal processes necessary for normal placenta development. The inflammatory response trigged by COVID-19 disease is very similar to that one found in preeclampsia pregnancies suggesting a possible link between SARS-CoV-2 infection and preeclampsia onset during pregnancy.

**Conclusion:**

Some studies showed that pregnancies affected by COVID-19 had higher incidence of preeclampsia compared with SARS-CoV-2-negative ones. However, increased blood pressure found in COVID-19 pregnancies does not allow to associate COVID-19 to preeclampsia as hypertension is a common factor to both conditions. At present, no diagnostic tools are available to discriminate real preeclampsia from preeclampsia-like syndrome in patients with SARS-CoV-2 infection. Thus, new specific diagnostic tools are necessary to assure an appropriate diagnosis of preeclampsia in these patients, especially in case of severe COVID-19 disease.

## INTRODUCTION

Severe acute respiratory syndrome coronavirus 2 (SARS-CoV-2) is the cause of coronavirus infectious disease 2019 (COVID-19) that was initially widespread in Wuhan (China) in December 2019 and then rapidly spread worldwide. It stressed the health care systems of many countries and caused hundreds of thousand deaths worldwide [1–3]. The main target of the virus is the respiratory system although many studies showed numerous other organs and tissues susceptible to SARS-CoV-2 infection, such as liver, testicle, kidney, nervous system, blood vessels, heart and placenta [4–6].

Placenta is a temporary and fundamental organ keeping mother and foetus in contact during gestation, it produces hormones, absorbs nutrients, and regulates gas exchange [7]. Placentation is a very important and tightly regulated process where extravillous trophoblast invades uterine spiral arteries forming vascular sinuses that provide nutrition to the foetus [8]. Impairment of this process can lead to gestational disorders, such as preeclampsia [9]. Preeclampsia is one of the four hypertensive disorders of pregnancy (HDP) categories that include chronic hypertension, preeclampsia–eclampsia, gestational hypertension and chronic hypertension with superimposed preeclampsia. HDP are among the leading causes of maternal and foetal morbidity and mortality. Thus, a proper diagnosis is crucial to initiate appropriate treatments and reduce the potential harm to the mother and the foetus. The different HDP are diagnosed according to hypertension onset, duration, severity and to different biochemical parameters [10]. Preeclampsia is a multisystem disorder that occurs in 2–8% of pregnancies worldwide; it is clinically characterized by proteinuria, hypertension during pregnancy and by a poor placenta differentiation [11]. Untreated preeclampsia can cause haemolysis, increased liver enzyme levels, low platelet count (HELLP syndrome), eclampsia. Thus, development and research of predictive preeclampsia markers play a key role in identifying this disorder early to avoid maternal and foetal health problems [12–14].

The angiotensin-converting enzyme 2 (ACE-2), a key regulator of blood pressure through the renin–angiotensin–aldosterone system, acts as cell receptor of Spike 1 protein (S1), which is a key component of the SARS-CoV-2 infection pathway [15]. Two forms of ACE-2 protein are detected: the full-length ACE2 form (called mACE-2), a type I integral membrane protein and the soluble form of ACE2 (called sACE-2) lacking the anchoring site and circulating in small amounts in the blood. sACE-2 is shed in the blood by the action of different proteases, such as metalloproteinase domain-containing protein (ADAM)-10, ADAM-17 and transmembrane protease serine 2 (TMPRSS2). mACE-2 acts as receptor site for S1 protein located on the envelope of SARS-CoV-2 allowing SARS-CoV-2 entry into the host cell [16]. Thus, SARS-CoV-2 can potentially infect any cell-expressing ACE-2 protein because of the high-affinity interaction between ACE-2 and S1 protein [17], inducing inflammation as response to virus [18].

Interestingly, it has been found that ACE-2 was also expressed in syncytiotrophoblast (STB), villous cytotrophoblast (CTB), extravillous trophoblastic cells (EVTs) and in human foetal membranes making this organ receptive to SARS-CoV-2 infection [5,19,20].

It has been found that SARS-CoV-2 can colonize multiple compartments of the maternal–foetal interface, in particular, the extravillous trophoblast in the basal plate. Moreover, virus was also found in foetal macrophages of placental foetal vessels, in the amniotic epithelial cells of the foetal membrane and in the tunica adventitia of the umbilical cord vessels. Interestingly, ACE-2 was downregulated in SARS-CoV-2 infected placenta resulting in upregulation of angiotensin II type-1 receptor autoantibodies (AT1-AA), and soluble Fms-like tyrosine kinase-1 (sFlt1) serum levels. Moreover, SARS-CoV-2 infected human placental cell line (JEG3) showed angiotensin II type 2 receptor (AT2R) and placental growth factor (PlGF) downregulation as well as angiotensin II type 1 receptor (AT1R) mRNAs upregulation. Thus, SARS-CoV-2 infection caused an imbalance in renin–angiotensin–aldosteorne system (RAAS) pathway diverting to AT1R arm proinflammatory way. In summary, SARS-CoV-2 can colonize ACE-2-expressing maternal and foetal cells in the placenta impairing RAAS signalling, a key pathway involved in regulating blood pressure [21], and leading to adverse hemodynamic outcomes like preeclampsia [5]. It has also been shown that soluble recombinant human ACE-2 prevents SARS-CoV-2 infection as it binds S1 protein present on the viral envelope blocking in part the binding between viral S1and cell surface ACE-2 [22]. Interestingly, plasma levels of ACE, ACE-2, and angiotensin-(1–7) were significantly higher in normal pregnancies compared with nonpregnant women and were lower in women affected by RAAS compared with healthy pregnancies [23]. Thus, it can be inferred that the high levels of sACE-2 detected in normal pregnancies may protect women from SARS-CoV-2 infection. On the contrary, low levels of sACE-2 found in pregnant women affected by preeclampsia might increase their susceptibility to SARS-CoV-2 infection.

Although COVID-19 is primarily a respiratory infection, it also has systemic effects that may resemble preeclampsia [24]. In fact, transcriptomic analyses showed that the maternal–foetal interface of SARS-CoV-2-infected women exhibited an increased activation of natural killer (NK) and T cells, markers also associated with pregnancy complicated by preeclampsia [19]. Moreover, it has been reported that SARS-CoV-2 infection causes an excessive inflammatory response that leads to the production of high levels of proinflammatory cytokines, such as IL-6, IL-2, IL-1β, TNF-α, and IFN-γ [18]. These cytokines are also increased in preeclampsia pregnancies [25] and this can be explained by the massive presence of T lymphocytes and macrophages in the placental intervillous space of patients with COVID-19 [26].

Patients affected by SARS-CoV-2 infection can develop different grades of COVID-19 severity according to the symptoms and clinical features: mild grade (e.g. fever, cough, or change in taste or smell), moderate grade (clinical/radiographic evidence of lower respiratory tract disease but SpO_2_ ≥94%), severe grade (SpO_2_ <94%, respiratory rate >30 breaths/min or lung infiltrates >50%) and critical grade (respiratory failure, shock, and multiorgan dysfunction or failure) [27].

A comparative morphological study of Shchegolev and colleagues evaluated the number of syncytial knots and vascular endothelial growth factor (VEGF) expression in placental villi of women with SARS-CoV-2 infection at different grades of disease severity. Interestingly, increased numbers of villous syncytial knots, high VEGF expression in syncytiotrophoblast and endothelial cells were found in infected placentas. These features are in common with those of pregnant women affected by preeclampsia and they have suggested the preeclampsia syndrome onset. Moreover, the number of syncytial knots and VEGF expression level in placental villi of pregnancy with COVID-19 increased with disease severity highlighting a morphological correlation between SARS-CoV-2 infection and preeclampsia [28].

Thus, the aim of this review is to analyse the current knowledge of COVID-19 in pregnancy-related to preeclampsia or preeclampsia-like syndrome onset in order to point out the possible links between SARS-COV-2 infection and preeclampsia/preeclampsia-like syndrome during pregnancy.

## SOURCES

We performed a systematic search using the databases PubMed and Web of Science up to 31 January 2022. The following keywords and their combination were used: SARS-COV-2, COVID-19, Severe Acute Respiratory Syndrome Coronavirus 2, Preeclampsia, PE, Preeclamp∗. Original studies conducted in humans evaluating the role of SARS-COV-2 infection in preeclampsia onset were the inclusion criteria used in this review. Review articles, systematic review, meta-analysis, case reports, and non-English articles were not included.

**FIGURE 1:**
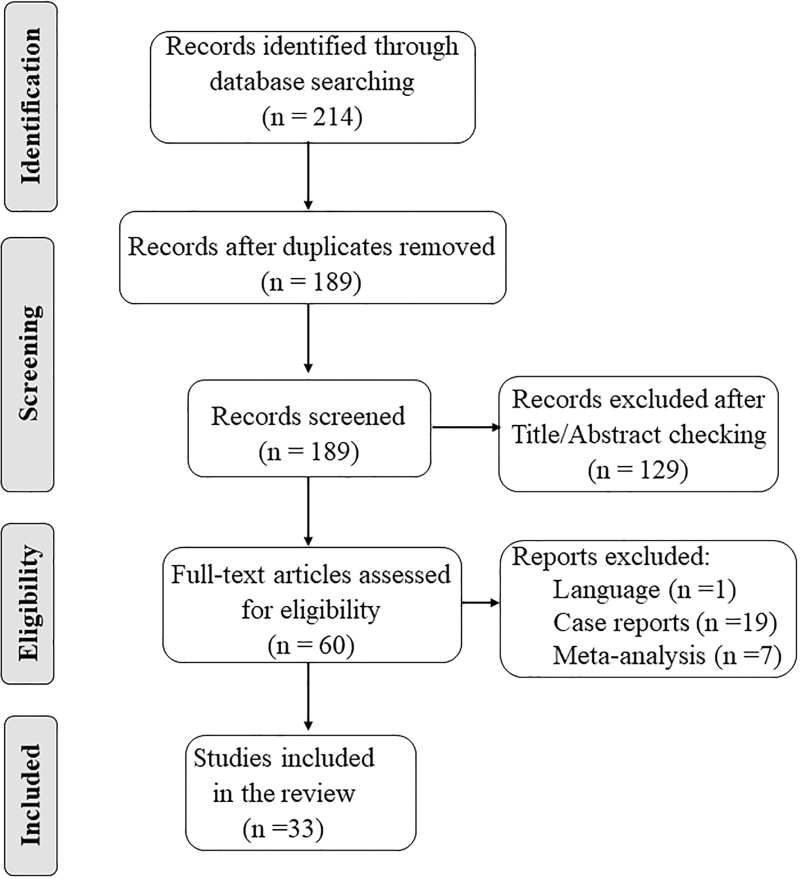
Flow diagram illustrating the article selection process discussed in this review.

Bibliographic research was conducted independently by two authors (G.T. and N.d.M.) via manual screening of the titles and abstracts of retrieved articles, any disputes were settled by a third author (D.M.). After the preliminary screening, full text of the selected articles was checked for eligibility. This study follows the Preferred Reporting Items for Systematic Reviews and Meta-Analyses (PRISMA) guideline [29] and the study selection process is shown in a flow diagram (Fig. 1).

## IS THERE A CORRELATION BETWEEN SEVERE ACUTE RESPIRATORY SYNDROME CORONAVIRUS 2 INFECTION AND PREECLAMPSIA ONSET?

### Studies finding no correlation between coronavirus 2019 and preeclampsia onset

An interesting retrospective case–control study compared ultrasound and Doppler findings of 103 negative and 106 positive pregnancies complicated by COVID-19 (57 asymptomatic, 19 severe and 30 mild disease). Although the authors did not find any differences in abnormal foetal ultrasound and Doppler findings, they found an increased incidence of preterm delivery and a trend (not significant) towards a higher prevalence of preeclampsia in SARS-CoV-2-positive pregnant women suggesting that Doppler test alone may not be appropriate to discriminate preeclampsia in SARS-CoV-2-positive pregnant women [30]. Moreover, Adhikari and colleagues performed a retrospective cohort study of 3374 pregnant women (252 positives and 3122 negatives for SARS-CoV-2 infection) and found no association between COVID-19 and pregnancy complications, such as preterm birth, preeclampsia and birth by caesarean section. However, 95% of positive patients had asymptomatic or mild disease and the incidence of preeclampsia has not been evaluated according to the COVID-19 severity [31]. However, these findings were in agreement with the following six studies. First, Cosma and colleagues evaluated asymptomatic/mild SARS-CoV-2 infection impact on preeclampsia and preterm birth comparing SARS-CoV-2-positive (*n* = 21) and SARS-CoV-2-negative (*n* = 81) pregnant women but no significant correlation was found suggesting that SARS-CoV-2 infection during pregnancy does not increase the risk of pregnant disorder development [32].

Second, a comparative study enrolling 199 pregnant women (66 COVID-19 and 133 normal pregnancies) found a significant increase in caesarean section rate in SARS-CoV-2-infected pregnant women but no differences in preeclampsia onset [33]. Unfortunately, these authors have not analysed the different grades of disease severity in these patients.

Third, an interesting morphological study enrolling 77 pregnant women affected by COVID-19 (87% asymptomatic) and 56 normal pregnancies found that pregnant women with COVID-19 infection were more likely to have foetal vascular malperfusion because of the presence of avascular placental villi and mural fibrin deposition suggesting that SARS-CoV-2 infection during pregnancy is a cause of placental histopathologic abnormalities even among asymptomatic patients. No difference was found in term of preeclampsia onset [34]. Fourth, a retrospective study enrolling 335 pregnant women (56 COVID-19 positives and 279 COVID-19 negatives) found no differences in obstetric outcomes between SARS-CoV-2-positive and negative cohorts. However, most SARS-CoV-2 positive patients (48/56) were asymptomatic and only 8/56 were symptomatic (6 mild and 2 severe COVID-19) [35].

Fifth, a retrospective study including 167 pregnant women with confirmed SARS-CoV-2 infection investigated the incidence of adverse pregnancy outcomes including preterm birth, preeclampsia, FGR, gestational diabetes mellitus at different gestational ages (10 first trimester, 28 second trimester and 129 third trimester). In this study, no significant difference on the incidence of these disorders among the three gestation groups was found suggesting that no significant correlation between the gestational age at the time of SARS-CoV-2 infection and adverse pregnancy frequency outcomes were present [36]. Sixth, a prospective observational study involving 121 pregnancies (16 COVID-19 positives and 105 COVID-19 negatives) in the first trimester of gestation detected that asymptomatic or mildly symptomatic SARS-CoV-2 positive women did not show significant increase of pregnancy complications compared with SARS-CoV-2 negative women [37] accordingly with previous study [36].

In summary, these studies (see Table 1) suggest that COVID-19 disease is not involved in preeclampsia onset but unfortunately they have considered patients mainly asymptomatic or mild symptomatic excluding the most severe COVID-19 disease as well as the relation with preeclampsia.

**TABLE 1:** Studies finding no correlation between severe acute respiratory syndrome coronavirus 2 infection and preeclampsia onset

		Gravidity of COVID-19 disease					
Type of study	Negative	Asymptomatic	Mild	Moderate	Severe	Results	Ref.
Retrospective (*n* = 209)	103	57	30	0	19	No differences in abnormal foetal ultrasound and Doppler findings. Increased incidence of preterm delivery and a trend (not significant) towards a higher prevalence of preeclampsia in SARS-CoV-2-positive pregnant women	[30]
Retrospective (*n* = 3374)	3122	239		13		No association between COVID-19 and adverse perinatal outcome, such as preterm birth, preeclampsia and caesarean birth	[31]
Case–control (*n* = 102)	81	21				No correlation was found between SARS-CoV-2 infection and preeclampsia onset. However, 80% of patients were SARS-CoV-2-negative and preeclampsia, HELLP syndrome and FGR were grouped together even if clinically very different	[32]
Comparative (*n* = 199)	133	66				Increased rate of caesarean section in pregnant women infected by SARS-CoV-2 but no differences in preeclampsia onset	[33]
Retrospective (*n* = 133)	56	67	10			Increased avascular villi and mural fibrin deposition in positive patients even if asymptomatic. No difference was found in term of preeclampsia onset. However, the 87% of patients studied were asymptomatic	[34]
Retrospective (*n* = 335)	279	48	6	0	2	No differences in obstetric outcomes between the SARS-CoV-2 positive and negative cohorts	[35]
Retrospective (*n* = 167)	0	167				No significant difference on the incidence of preterm birth, preeclampsia, FGR and gestational diabetes mellitus among the three gestation groups was found	[36]
Prospective (*n* = 121)	105	16		0	0	Asymptomatic or mildly symptomatic women during the first trimester of pregnancy did not show significantly increase of pregnancy complications compared to SARS-CoV-2-negative women	[37]

COVID-19, coronavirus disease 2019; FGR, Foetal Growth Restriction; HELLP syndrome, Hemolysis, Elevated Liver enzymes and Low Platelets syndrome; SARS-CoV-2, severe acute respiratory syndrome coronavirus 2.

### Studies finding a correlation between coronavirus disease 2019 and preeclampsia onset

Twenty-six studies found a statistical correlation between COVID-19 in pregnancy and preeclampsia onset but not all evaluated COVID-19 disease severity.

#### Studies not evaluating coronavirus disease 2019 disease severity

In literature, not all the studies on pregnant women have detected a correlation between the risk to preeclampsia development and COVID-19 severity. In fact, the following studies although founding a correlation between COVID-19 and preeclampsia onset did not evaluate COVID-19 disease severity. However, they suggested that SARS-CoV-2-infected pregnant women are at higher risk to develop preeclampsia.

Two different types of study enrolling a total of 3583 COVID-19-positive and 338 647 COVID-19-negative pregnant women found that preeclampsia, preterm birth and caesarean delivery were significantly higher in SARS-CoV-2 infected pregnant women [38,39]. Moreover, Litman *et al.*[40] in an observational cohort study including 2708 COVID-19 positive and 39 562 COVID-19-negative pregnancies found that the first group was more likely to have preeclampsia experience, early and late preterm birth. These data are in agreement with four studies [41–44] that have compared a total of 1130 SARS-CoV-2 positive with 861 945 SARS-CoV-2 negative pregnant women showing that SARS-CoV-2 positive pregnant women were more likely to develop preeclampsia during pregnancy. In addition, the incidence of preterm labour and premature rupture of membrane (PROM), a breakage of the amniotic sac before the onset of labour, was higher in pregnant women affected by COVID-19 compared with the negative ones [43,44]. Moreover, placental SARS-CoV-2 presence was more frequent among women with preeclampsia and high placental viral load was associated with severe preeclampsia. So, SARS-CoV-2 infection could trigger gestational hypertensive disorders and the persistent placental infection could lead to placental damage [42].

Interestingly, some diseases (such as diabetes, autoimmune diseases, chronic respiratory conditions, cancer and asthma) may also influence preeclampsia onset in pregnant women affected by COVID-19 as reported in a case–control study that compared 49 cases (pregnant women with underlying diseases) and 49 controls. In this study, it has been shown that preterm labour, preeclampsia, and eclampsia were significantly higher in cases compared with controls suggesting a close monitoring of pregnant women with underlying diseases as these can be considered risk factors to comorbidity development, such as preeclampsia [45].

A summarizing table of the studies previously mentioned is shown in Table 2.

**TABLE 2:** Studies founding correlation not evaluating coronavirus disease 2019 disease severity

Type of study	COVID-19 Positive	COVID-19 Negative	Results	Ref.
Retrospective (*n* = 342 080)	3527	338 553	Preeclampsia, preterm birth, foetal death and birth by caesarean delivery were significantly higher in pregnant women with SARS-CoV-2 infection	[38]
Prospective (*n* = 150)	56	94	Pregnant women with COVID-19 had an increased risk of preeclampsia, preterm labour and caesarean section	[39]
Observational (*n* = 42 270)	2708	39 562	Women with COVID-19 were more likely to experience early, late preterm birth and preeclampsia compared with women without COVID-19	[40]
Case-control (*n* = 759)	155	604	Pregnant women testing positive to SARS-CoV-2 infection were more likely to have preeclampsia	[41]
Case-control (*n* = 28)	14	14	SARS-CoV-2 presence in placenta was more frequent in women affected by preeclampsia and high placental viral load was associated with severe preeclampsia.	[42]
Retrospective (*n* = 261)	87	174	Preterm labour, preeclampsia, and premature rupture of membrane (PROM) incidences were higher in pregnant women affected by COVID-19	[43]
Retrospective (*n* = 244 645)	874	243 771	Women with COVID-19 had a high frequency of preeclampsia/eclampsia, gestational hypertension, preterm labour and caesarean section	[44]

COVID-19, coronavirus disease 2019; SARS-CoV-2, severe acute respiratory syndrome coronavirus 2.

**TABLE 3:** Studies with correlation between preeclampsia onset and coronavirus disease 2019 disease severity

		COVID-19 disease severity					
Type of study	Negative	Asymptomatic	Mild	Moderate	Severe	Results	Ref.
Prospective (*n* = 42)	0	34			8	Increased incidence of preeclampsia/preeclampsia-like syndrome in pregnancies complicated by severe COVID-19 disease.	[24]
Retrospective case-control (*n* = 240)	170	60	9	1	0	Preeclampsia incidence was higher in positive women than in negative ones	[46]
Retrospective (*n* = 1649)	1351	290	0	8	0	Significant higher percentage of placental fibrinoid in women with low CTs (<25). preeclampsia onset associated with symptomatology but not with risk factors or CT values.	[47]
Retrospective (*n* = 1650)	1498	143	0	0	9	Increased rates of PROM, preterm delivery, gestational hypertension and preeclampsia without severe features (eclampsia, and HELLP syndrome) in positive patients	[48]
Retrospective (*n* = 813)	760	38	7	2	6	Women with a COVID-19 diagnosis were significantly more likely to have preterm delivery and preeclampsia with severe features	[49]
Retrospective (*n* = 253)	0	227	0	0	26	Increased incidence of preeclampsia, premature rupture of membranes (PROM), high rate of prematurity and perinatal lethality in COVID-19 patients.	[50]
Retrospective (*n* = 1223)	0	696	417	72	38	Increased risk of preterm birth and preeclampsia with the increase of severity infection.	[51]
Retrospective (*n* = 1219)	0	579	326	173	43	Severe/critical COVID-19 disease, but not mild/moderate COVID-19 disease, increased the risk of caesarean section, preeclampsia and preterm birth compared with asymptomatic patients.	[52]
Retrospective (*n* = 617)	617	489		93	35	Disease severity was associated with age older than 35 years and obesity, as well as pre-existing diabetes, previous preeclampsia and gestational hypertension or preeclampsia. Preterm birth rate in women with severe and critical COVID-19 was higher than in nonsevere disease	[53]
Observational (*n* = 19)	0	0	14	0	5	Increased preeclampsia onset in pregnant women with severe COVID-19 disease requiring oxygen support	[54]
Prospective (*n* = 23)	0	0	13	2	8	COVID-19 was associated with high preterm delivery prevalence, preeclampsia and caesarean section compared with normal pregnancies. COVID-19 pregnant women were more likely to develop severe preeclampsia	[55]
Retrospective (*n* = 2184)	1459	292	433			SARS-CoV-2 infection during pregnancy was strongly associated with preeclampsia, especially among nulliparous women but this association was independent from disease severity (symptomatic vs. asymptomatic).	[56]
Comparative (*n* = 2130)	1424	288	418			Positive pregnant women were at high risk to develop preeclampsia/eclampsia during pregnancy and undergoing preterm delivery. Preeclampsia increased risk was also showed in asymptomatic women with COVID-19 diagnosis	[57]
Multicentric prospective (*n* = 2954)	1607	688	467		0	Increased preterm delivery risk in infected pregnant women while the risk of preeclampsia was similar in both infected and noninfected patients. Positive pregnant women ended gestation with severe preeclampsia whereas negative pregnant women with moderate preeclampsia. preeclampsia severity may be related to the infection status.	[58]
Multicentric observational (*n* = 1347)	1347	688	467		192	High ICU admittance rate in pregnancies obtained by IVF was attributed to preeclampsia due to infection and not to the method of conception	[60]

COVID-19, coronavirus disease 2019; HELLP syndrome, Hemolysis, Elevated Liver enzymes and Low Platelets syndrome; IVF, in-vitro fertilization; SARS-CoV-2, severe acute respiratory syndrome coronavirus 2.

#### Studies evaluating coronavirus disease 2019 disease severity

Four retrospective studies [46–49] including a total 3779 SARS-CoV-2 negative and 275 positive pregnancies (241 asymptomatic, 16 mild, three moderate and 15 severe) showed that SARS-CoV-2-positive women had significant higher gestational hypertension and preeclampsia rates compared with negative patients [46,48]. In addition, women with COVID-19 diagnosis had significantly more likely both preterm delivery and preeclampsia with severe features [49]. Moreover, as the SARS-CoV-2 infection is mainly diagnosed by molecular test (RT-qPCR), it has been found that preeclampsia was significantly associated with symptomatology but not with cycle threshold (CT) values reported by COVID-19 test. Interestingly, a high percentage of placental fibrinoids, suggesting a possible placenta damage, was found in women with low cycle threshold values (CT <25) [47] indicating a high viral load.

Other three retrospective studies [50–52] enrolling a total of 2597 COVID-19 positive pregnant women (1502 asymptomatic, 743 mild, 245 moderate and 107 severe) showed that the main co-morbidities detected in positive patients were preterm delivery, PROM, preeclampsia and caesarean section. Moreover, severe or critical COVID-19 diseases but not mild or moderate COVID-19 diseases, were associated with increased risk of caesarean section and preterm birth compared with asymptomatic patients [52]. Lai and colleagues found that preeclampsia onset rate was 1.9% in asymptomatic patients, 2.2% in patients with mild disease, 5.7% in moderate disease and 11.1% in severe disease. In addition, women with a severe COVID-19 disease had a five-fold greater risk of preeclampsia development compared with the asymptomatic women suggesting that infection severity has a key role in preeclampsia onset. Moreover, the risk of preeclampsia development in pregnant women with moderate or severe COVID-19 disease was 3.3-fold higher than in those with asymptomatic or mild infection and preterm birth risk was also increased with COVID-19 disease severity [51].

Other studies confirmed these correlations [53–55] but Osaikhuwuomwan *et al.* found that COVID-19 disease severity was associated also with mother age older than 35 years and obesity, as well as pre-existing diabetes and previous preeclampsia. In addition, preterm birth rate was higher in women with severe and critical COVID-19 disease compared with the nonsevere form of disease [54]. In addition, COVID-19-infected pregnant women showed an increased preterm labour risk and caesarean delivery compared with COVID-19-negative pregnancies [55].

Contrarily, two studies [56,57] found an increased preeclampsia incidence in asymptomatic SARS-CoV-2-positive pregnant women. In particular, Papageorghiou *et al.*[56] compared 292 asymptomatic, 433 symptomatic and 1459 negative pregnancies and found that SARS-CoV-2 infection during pregnancy was strongly associated with preeclampsia in both asymptomatic and symptomatic patients. These data were confirmed by another study involving 18 countries and enrolling 706 SARS-CoV-2 infected pregnant women and 1424 control pregnant women [57].

A multicentric prospective study enrolling a cohort of 1347 SARS-CoV-2-positive and 1607 negative pregnancies showed that preterm delivery risk was higher in positive patients while preeclampsia risk was similar in both groups. In addition, the authors highlighted that SARS-CoV-2-positive women developed severe preeclampsia whereas negative women developed moderate preeclampsia but severe preeclampsia cases in positive individuals may be overestimated as severe preeclampsia diagnosis, in addition to hypertension, is also based on clinical parameters that can be altered in COVID-19 disease, such as increase of liver enzymes, lactate-dehydrogenase (LDH) and thrombocytopenia [58].

Generally, in-vitro fertilization (IVF) pregnancy is considered a high-risk pregnancy because of increased clinical complications, such as preeclampsia and FGR [59]. A multicentric observational study evaluated totally 1347 SARS-CoV-2-infected pregnant women: 1273 spontaneous pregnancies and 74 IVF pregnancies. This study reported that hight ICU admittance rate of IVF pregnancies was because of preeclampsia but not to fertilization type suggesting that preeclampsia was caused by SARS-CoV-2 infection but not because of conception method [60].

All studies together suggest that the acute inflammation because of the SARS-CoV-2 infection may contribute, especially in severe disease, to increase hypertensive disorders, particularly preeclampsia. Moreover, COVID-19 can lead to other pregnancy complications, such as preterm delivery, PROM and caesarean section suggesting careful and continuous monitoring of SARS-CoV-2 positive pregnant patients.

## IS PREECLAMPSIA A RISK FACTOR FOR SEVERE ACUTE RESPIRATORY SYNDROME CORONAVIRUS 2 INFECTION OR PREECLAMPSIA IS DUE TO VIROSIS?

### Virosis as cause of preeclampsia

Endothelial cells of both venous and arterial vessels express ACE-2 and can be potential targets of SARS-CoV-2. Thus, SARS-CoV-2 infected endothelial cells can lead to endotheliitis, systemic vasculitis and disseminated intravascular coagulation (DIC) [61] explaining the high frequency of thrombosis and thromboembolism reported in COVID-19 patients [62].

Although it is known that one of the major causes of preeclampsia is superficial trophoblast invasion of uterine spiral arteries during the first trimester of gestation [9], an alteration of this process because of SARS-CoV-2 infection could be excluded as no differences in preeclampsia onset have been reported in positive women infected during the first trimester of pregnancy compared with second and third trimester of gestation [36]. Thus, we can hypothesize that preeclampsia syndrome in pregnancies complicated by SARS-CoV-2 infection could be because of endothelial dysfunctions caused by vasculitis and/or endotheliitis. In addition, it has been reported that the likelihood to develop preeclampsia significantly increases when diabetes is present before pregnancy [63]. In fact, it is known that endothelial inflammation and dysfunction are early events of vascular complications of diabetes that represents also a risk factor for preeclampsia onset. Therefore, endothelial dysfunction, common in diabetes and SARS-CoV-2 infection, could increase the risk to develop preeclampsia/preeclampsia-like syndrome [64,65].

### Preeclampsia as risk factor of severe acute respiratory syndrome coronavirus 2 infection

As we above discussed, low levels of sACE-2 have been found in pregnant women sera affected by preeclampsia compared with healthy pregnancies. As ACE-2 acts as receptor for SARS-CoV-2, high blood levels of sACE-2 in normal pregnancy may have a protective effect against SARS-CoV-2 infection as sACE-2 can block virus binding to mACE-2 avoiding virus cell entry and the consequent infection. Thus, low levels of sACE-2 in preeclampsia patients may be a risk factor to SARS-CoV-2 infection allowing virus cell entry through mACE-2 binding.

Moreover, it is known that preeclampsia is characterized by an excessive increase of pro-inflammatory cytokines that cause placental dysfunction and maternal systemic complications [25]. In particular, IL-6, IL-2, IL-1β, TNF-α and IFN-γ levels are generally increased in preeclampsia pregnancies and are also the major components of cytokine storm found in patients affected by COVID-19 [66]. Thus, preeclampsia with superimposed SARS-CoV-2 infection could worsen the inflammatory status of these pregnant women leading to more severe complications, such as ICU admittance and oxygen support as we previously discussed [54,60].

## ROLE OF ALDOSTERONE IN PREECLAMPSIA AND CORONAVIRUS DISEASE 2019

It is known that high aldosterone levels suppresses renin activity leading to hypertension and inflammation [67,68]. It has been reported that high aldosterone plasma levels are associated with severe COVID-19 disease suggesting a possible role of RAAS pathway in COVID-19 outcome [69] and in cytokine storm found in this disease [70]. Previous studies demonstrated an important role of angiotensin ATR1 receptors [71], aldosterone and primary hyperaldosteronism in preeclampsia onset. In particular, primary hyperaldosteronism is common in women who will develop preeclampsia during pregnancy [68,72,73]. At present, no data are available on aldosterone levels in pregnant women with preeclampsia/HPD syndrome affected by COVID-19. However, RAAS pathway is altered in both COVID-19 and preeclampsia so, high aldosterone levels could have the same role in both these disorders causing hypertension and inflammation.

## DISCUSSION

SARS-CoV-2 infection (symptomatic and asymptomatic) during pregnancy may cause many pregnancy complications, such as spontaneous abortion, FGR, premature delivery, caesarean section and hypertensive disorders including preeclampsia and preeclampsia-like syndrome. Some studies have shown that COVID-19 is a risk factor for the origin of preeclampsia; however, other studies did not find any correlation between preeclampsia and COVID-19. These contrasting results are because of the different cohorts analysed, cohort size and different variables analysed (presence/absence of symptoms, gravity of COVID-19 disease). In particular, only few studies considered different grades of COVID-19 severity and their correlation to preeclampsia. Severe symptomatic COVID-19 disease shares clinical and laboratory features similar to those reported in preeclampsia, such as hypertension, malaise, haemolysis, transaminitis, elevated LDH, elevated blood urea nitrogen/creatinine ratio and thrombocytopenia. Moreover, the inflammatory response trigged by SARS-CoV-2 infection is very similar to that found in preeclampsia pregnancies. In fact, it has been demonstrated that SARS-CoV-2 infection up-regulates vascular endothelial growth factor receptor 1 (VEGFR-1, also known as FLT1), endoglin (ENG, the main antiangiogenic factor involved in preeclampsia), vasoconstrictive peptides [Endothelin 1 (END1), urotensin 2 (UTS2), angiotensin 1 (AGT)], phosphodiesterase 5A (PDE5A; a nitric oxide modulator) and hypoxia-inducible factor 1-α (HIF-1α; a key factor involved in placental invasion and increased in preeclampsia) that are the main factors involved also in preeclampsia onset [74,75]. In fact, soluble FLT1 and ENG antagonizes VEGF binding to its receptor contributing to hypertension, proteinuria and endothelial cell dysfunction in preeclampsia [76]. This mechanism could be present also in COVID-19 pregnancies causing increased blood pressure and endothelial dysfunction.

Generally, it has been reported that COVID-19 can increase SBP and DBP causing new hypertension onset in both males and females [77,78]. In addition, it has been also described that pregnancies affected by COVID-19 had higher incidence of gestational hypertension compared with COVID-19-negative pregnancies [42,44,57]. Thus, increased blood pressure found in COVID-19 pregnancies does not allow to associate COVID-19 to preeclampsia/HDP as hypertension is a common factor to both conditions. At present, preeclampsia and preeclampsia-like syndrome associated to the viral infection can be discriminated at delivery. In particular, preeclampsia will begin to resolve after delivery while preeclampsia-like syndrome because of COVID-19 is unrelated to delivery time and symptoms persist after delivery. Thus, scheduled caesarean section to resolve preeclampsia-like syndrome in COVID-19 pregnancies will not resolve the symptoms in these patients as differently from actual preeclampsia, symptoms are not because of gestational complications.

Summarizing the data analysed in this review, we can assume that new specific diagnostic tools are necessary to discriminate an actual preeclampsia in patients with SARS-CoV-2 infection, especially in severe disease, in order to assure proper treatments.

## ACKNOWLEDGEMENTS

Author contributions: G.T. – literature search, conceptualized the paper, wrote the paper, and edited the final version of paper, contributed to figures. S.F. –critical evaluation and feedback towards improving the manuscript. N.d.M. – literature search, edited the final version of the paper. G.B. – critical evaluation and feedback towards improving the manuscript. A.C. –critical evaluation and feedback towards improving the manuscript. S.R.G. – writing- reviewing and editing. D.M. – writing- reviewing and editing, supervision, contributed to figures.

Funding: G.T. is a recipient of a fellowship Starting Grant 2018 (SG-2018-12367994) of the Italian Ministry of Health.

### Conflicts of interest

There are no conflicts of interest.
